# Bioinformatical Analysis of miRNA-mRNA Interaction Network Underlying Macrophage Aging and Cholesterol-Responsive Difference between Young and Aged Macrophages

**DOI:** 10.1155/2020/9267475

**Published:** 2020-06-12

**Authors:** Jianqing Li, Xue Yin, Bingyu Zhang, Chen Li, Peirong Lu

**Affiliations:** Department of Ophthalmology, the First Affiliated Hospital of Soochow University, 188 Shizi Street, Suzhou 215006, China

## Abstract

**Purpose:**

Macrophage aging is involved with the occurrence and progression of age-related macular degeneration (AMD). The purpose of this study was to identify the specific microRNAs (miRNA), mRNAs, and their interactions underlying macrophage aging and response to cholesterol through bioinformatical analysis in order to get a better understanding of the mechanism of AMD.

**Methods:**

The microarray data were obtained from Gene Expression Omnibus (accession GSE111304 and GSE111382). The age-related differentially expressed genes in macrophages were identified using R software. Further miRNA-mRNA interactions were analyzed through miRWalk, mirTarBase, starBase, and then produced by Cytoscape. The functional annotations including Gene Ontology and KEGG pathways of the miRNA target genes were performed by the DAVID and the STRING database. In addition, protein-protein interaction network was constructed to identify the key genes in response to exogenous cholesterol.

**Results:**

When comparing aged and young macrophages, a total of 14 miRNAs and 101 mRNAs were detected as differentially expressed. Besides, 19 validated and 544 predicted miRNA-mRNA interactions were detected. Lipid metabolic process was found to be associated with macrophage aging through functional annotations of the miRNA targets. After being treated with oxidized and acetylated low-density lipoprotein, miR-714 and 16 mRNAs differentially expressed in response to both kinds of cholesterol between aged and young macrophages. Among them, 6 miRNA-mRNA predicted pairs were detected. The functional annotations were mainly related to lipid metabolism process and farnesyl diphosphate farnesyl transferase 1 (FDFT1) was identified to be the key gene in the difference of response to cholesterol between aged and young macrophages.

**Conclusions:**

Lipid metabolic process was critical in both macrophage aging and response to cholesterol thus was regarded to be associated with the occurrence and progression of AMD. Moreover, miR-714-FDFT1 may modulate cholesterol homeostasis in aged macrophages and have the potential to be a novel therapeutic target for AMD.

## 1. Introduction

Macrophages, being critical cells of the innate immune system, play significant roles in development, homeostasis, immunity, and tissue repair [1]. Nevertheless, aged macrophages have been generally reported to exhibit functional changes such as reduced phagocytosis [2], increased angiogenesis [3], and impaired cholesterol metabolism [4]. Impairment in cholesterol homeostatic mechanism has been regarded to be associated with some diseases of the elderly, such as atherosclerosis [5] and age-related macular degeneration (AMD) [6].

AMD is a progressive disease of the central retina and a leading cause of vision loss worldwide [7]. AMD is initially characterized by accumulation of lipid-rich deposits known as drusen, which is a risk factor of the disease progression into late AMD [8]. However, the role of macrophages in cholesterol homeostasis in the pathogenesis of AMD remains elusive. With the development of anti-VEGF therapies [9], treatments for wet AMD have been largely evolved. However, because anti-VEGF agents have some adverse events [10] and do not address early AMD and the process of progression to late AMD [11], there is an urgent need for new therapeutic options for AMD. Therefore, a better understanding of the pathological mechanism of the disease development and progression is required for the development of new treatments.

MicroRNAs (miRNAs) are small noncoding RNAs that can regulate the expression of multiple mRNAs [12]. Identification of miRNA-mRNA interactions can be performed through computational methods [13, 14] and is beneficial to the understanding of the gene-regulatory role of miRNAs in the therapeutic role of mRNAs.

In this study, we identified the impact of senescence on macrophages as well as the difference in cholesterol response between aged and young macrophages regarding the differential expression of miRNAs, mRNAs. Further analysis of miRNA-mRNA interactions and functional annotation of the miRNA target genes were performed to understand the molecular basis and the related pathways. At last, protein-protein interaction (PPI) network was analyzed to identify the key genes in response to exogenous cholesterol. We sought to study the roles of macrophages in cholesterol modulation in order to find a potential therapeutic method for AMD.

## 2. Methods

### 2.1. Datasets

The miRNA expression dataset GSE111304 [15] and the mRNA expression dataset GSE11382 [16] were obtained from the Gene Expression Omnibus (GEO, http://www.ncbi.nlm.nih.gov/geo/). The profile of GSE111304 was based on the platform of GPL16384 [miRNA-3] Affymetrix Multispecies miRNA-3 Array, and the platform of GSE111382 was GPL6246 [MoGene-1_0-st] Affymetrix Mouse Gene 1.0 ST Array [transcript (gene) version]. The miRNA and mRNA expressions were profiled on aged (18-month-old) and young (2- to 3-month-old) peritoneal macrophages, which were obtained from wild type C57BL/6J mice and then left untreated, treated with 25 *μ*g/ml oxidized low-density lipoprotein (ox-LDL) for 24 hours or treated with 25 *μ*g/ml acetylated low-density lipoprotein (ac-LDL) for 24 hours.

### 2.2. Identify Differentially Expressed miRNAs and mRNAs

The raw data of miRNA and mRNA microarray were interpreted by limma package (limma, http://www.bioconductor.org/packages/release/bioc/html/limma.html) of R software (version 3.5.1) [17] to identify the differentially expressed miRNAs and mRNAs. Expression comparison was conducted by Student's *t*-test and the thresholds were ∣log (fold change) | >1 and *p* value <0.05.

### 2.3. miRNA-mRNA Interaction Analysis

We applied miRWalk (http://mirwalk.umm.uni-heidelberg.de/) [18], miRTarBase (http://miRTarBase.mbc.nctu.edu.tw/) [19] and starBase (http://starbase.sysu.edu.cn/starbase2/) [20, 21] to conduct in silico prediction of miRNA targets and visualize the interaction data through Cytoscape [22].

The first step was to identify miRNA targets that have previously been validated by experimental approaches through these three data resources.

Next, predicted miRNA-mRNA targets were detected by miRWalk and the other tools available in that website, including TargetScan [23], miRanda [24], and RNA22 [25]. mRNAs that could be predicted in all four databases were defined as highly predicted miRNA targets.

### 2.4. Functional Annotations of miRNA Target Genes

For those mRNA targets, Gene Ontology (GO) and Kyoto Encyclopedia of Genes and Genomes (KEGG) pathway analysis were conducted through the Database for Annotation, Visualization, and Integrated Discovery (DAVID) (https://david.ncifcrf.gov/) [26, 27].

### 2.5. PPI Network Construction

For cholesterol-responsive miRNA targets, PPI analysis was performed through the Search Tool for the Retrieval of Interacting Genes/Proteins (STRING) database (http://www.string-db.org) and produced by Cytoscape [22].

## 3. Results

### 3.1. Differentially Expressed miRNAs and mRNAs in Macrophage Aging

**Figure 1:**
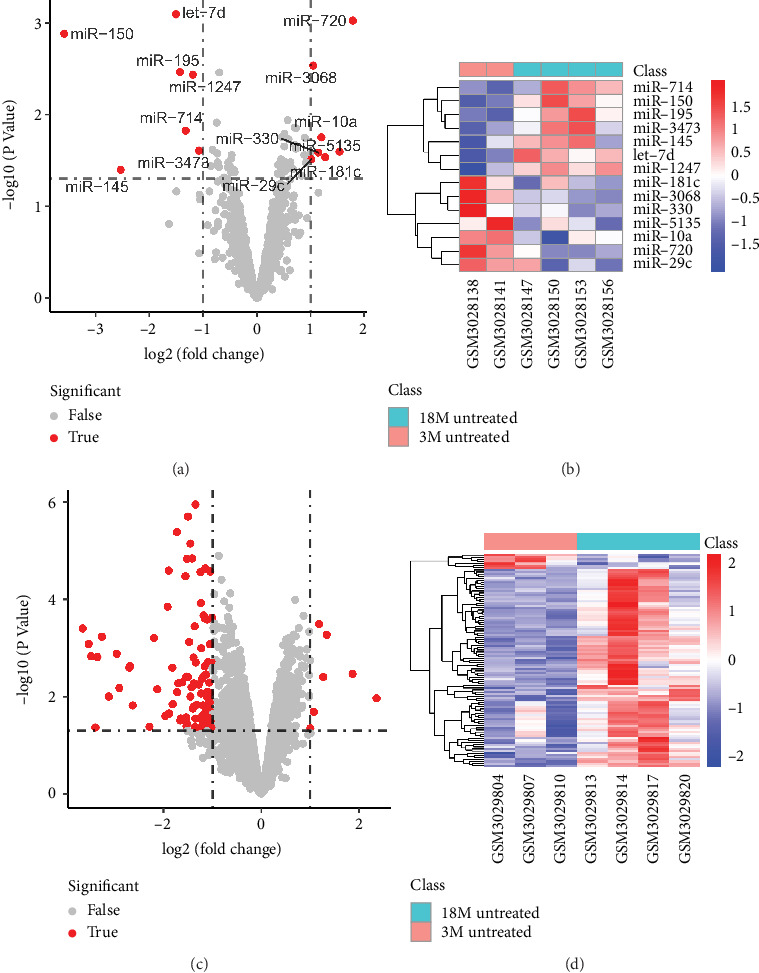
Differentially expressed miRNAs and mRNAs in macrophage aging. The volcano plot (a) and the heat map (b) showed that a total of 14 miRNAs were detected to be differentially expressed; 7 were upregulated and 7 downregulated. The volcano plot (c) and the heat map (d) displayed that 101 miRNAs expressed differentially between aged and young macrophages, and 7 of them were upregulated.

To determine the differentially expressed miRNAs and mRNAs in aged macrophage, we compared the profiles of aged and young macrophages that were remained untreated. A total of 14 miRNAs and 101 mRNAs were detected as differentially expressed. The volcano plots and heat maps were displayed in Figure 1.

### 3.2. miRNA-mRNA Interactions Underlying Macrophage Aging

**Figure 2:**
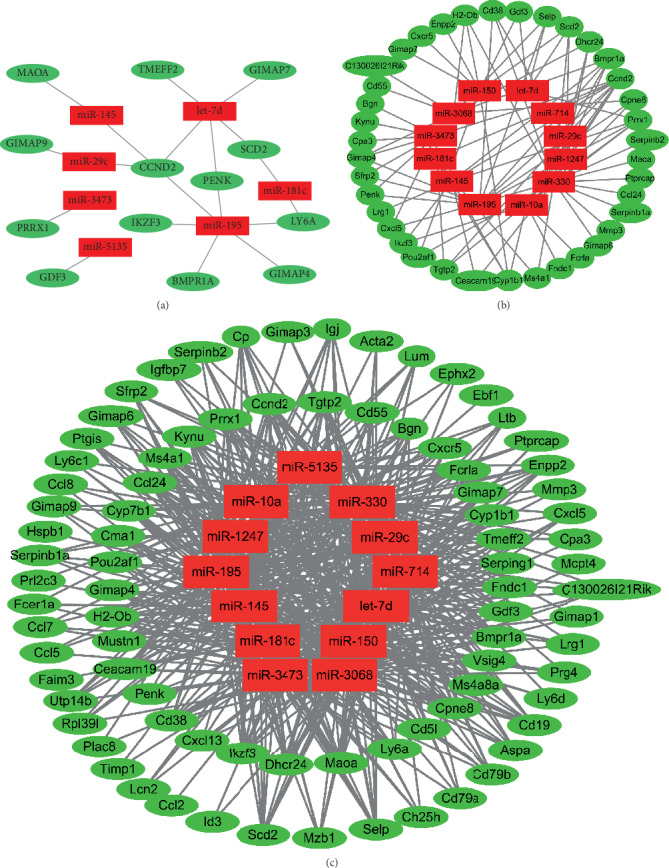
miRNA-mRNA interactions underlying macrophage aging. miRNA-mRNA interaction analysis was conducted on the differentially expressed miRNAs and mRNAs in macrophage aging and 19 validated (a) and 544 predicted pairs (b) were identified. In addition, 83 highly predicted miRNA-mRNA pairs (c) were found which could be detected by four prediction databases.

Among these differentially expressed miRNAs and mRNAs, a total of 19 validated miRNA-mRNA interactions were identified (Figure 2(a)). In addition, 544 predicted interactions were detected, involving 13 miRNAs and 84 mRNAs (Figure 2(b)). When it comes to the highly predicted miRNA targets, 83 miRNA-mRNA interactions were obtained (Figure 2(c)), which involves 12 miRNAs and 37 mRNAs.

### 3.3. Functional Annotations of Age-Related miRNA Target Genes

GO analysis of the validated and predicted miRNA targets was conducted, and a total of 65 biological processes (BP), 14 molecular functionings (MF), and 9 cellular components (CC) were identified in DAVID. In addition, 7 KEGG pathways were detected. The top 9 GO and the KEGG pathways were displayed in Table 1. Lipid metabolic process is one of the top 9 BP, and the rest were immune response, inflammatory response, chemotaxis, positive regulation of angiogenesis, oxidation-reduction process, chemokine-mediated signaling pathway, cellular response to interleukin-1, and positive regulation of cell proliferation.

### 3.4. Cholesterol-Responsive Differentially Expressed miRNAs and mRNAs

We separately analyzed differentially expressed miRNAs and mRNAs in young and aged macrophages when treated with oxLDL or acLDL to study the different response of these cells to exogenous cholesterol.

**Figure 3:**
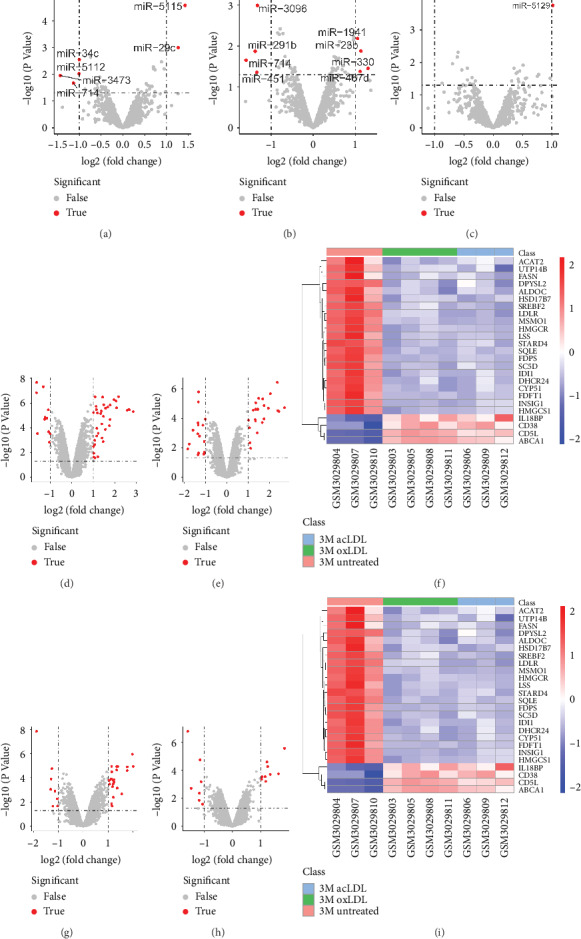
Cholesterol-responsive differentially expressed miRNAs and mRNAs between aged and young macrophages. In young macrophages, 6 miRNAs were differentially expressed in response to (oxLDL) (a) and 8 miRNAs in response to (acLDL) (b). Altogether, only miR-714 was downregulated in response to both acLDL and oxLDL. In aged macrophages, no differentially expressed miRNA was identified in response to oxLDL, and miR-5129 was the only differentially upregulated miRNA in response to acLDL (c). Therefore, miR-714 was the differentially expressed miRNAs between young and aged macrophage's response to cholesterol. With regards to differentially expressed mRNAs, 47 were detected when treated with oxLDL (d) and 39 with acLDL (e) in young macrophages and 25 mRNAs expressed differentially in response to both oxLDL and acLDL (f). In aged macrophages, 30 and 16 mRNAs expressed differentially in response to oxLDL (g) and acLDL (h), respectively, and 13 mRNAs in response to both (i).

In young macrophages, only miR-714 was downregulated in response to both acLDL and oxLDL, though 6 and 8 miRNAs were differentially expressed in response to oxLDL (Figure 3(a)) and acLDL (Figure 3(b)), respectively. In aged macrophages, no differentially expressed miRNA was identified in response to oxLDL, and miR-5129 was the only differentially upregulated miRNA in response to acLDL (Figure 3(c)). Hence, the differentially expressed miRNAs between young and aged macrophage's response to exogenous cholesterol were miR-714.

47 differentially expressed mRNAs were detected in response to exogenous oxLDL in young macrophages (Figure 3(d)), and 39 were found differentially expressed in response to acLDL (Figure 3(e)). Among them, 25 mRNAs were identified differentially expressed in response to both oxLDL and acLDL, with 21 mRNAs downregulated and 4 upregulated (Figure 3(f)). In aged macrophages, 30 mRNAs expressed differentially in response to oxLDL (Figure 3(g)), and 16 mRNAs expressed differentially in response to acLDL (Figure 3(h)). A total of 13 mRNAs were identified differentially expressed in response to both kinds of exogenous cholesterol, 9 and 4 being down- and upexpressed, respectively (Figure 3(i)). By comparing the 25 cholesterol-responsive mRNAs in young macrophages and the 13 mRNAs in aged ones, a total of 16 mRNAs were found to differentially expressed between young and aged macrophages in response to exogenous cholesterol.

### 3.5. miRNA-mRNA Interactions of Cholesterol-Responsive Difference between Young and Aged Macrophages

**Figure 4:**
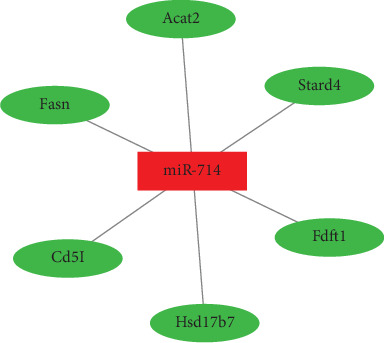
miRNA-mRNA interactions of cholesterol-responsive difference between aged and young macrophages. miRNA-mRNA interaction identification was conducted on cholesterol-responsive differentially expressed miRNAs and mRNAs between aged and young macrophages, and 6 predicted pairs were identified.

Identification of miRNA-mRNA interactions was conducted on the differentially expressed miRNA and mRNAs between young and aged macrophage's response to exogenous cholesterol. No validated interaction was found; nevertheless, 6 miRNA-mRNA predicted pairs were detected, and they were all predicted by one or two databases (Figure 4).

### 3.6. Functional Annotations of Age-Related miRNA Target Genes in Response to Cholesterol

GO analysis of the cholesterol-responsive miRNA targets was conducted. In all, 12 BP and 2 MF were found through the String online database and were mainly lipid metabolism associate, including lipid metabolic process, cellular lipid metabolic process, small molecule metabolic process, steroid metabolic process, lipid biosynthetic process, small molecule biosynthetic process, oxidation-reduction process, cellular lipid biosynthetic process, cholesterol biosynthetic process, cholesterol metabolic process, lipid modification, fatty acid metabolic process, acetyltransferase activity, oxidoreductase activity, and acting on the CH-OH group of donors. In addition, the detected 3 KEGG pathways were all about lipid metabolism, including metabolic pathways, fatty acid metabolism, and steroid biosynthesis (shown in Table 2).

### 3.7. PPI Analysis of Age-Related miRNA Target Genes in Response to Cholesterol

**Figure 5:**
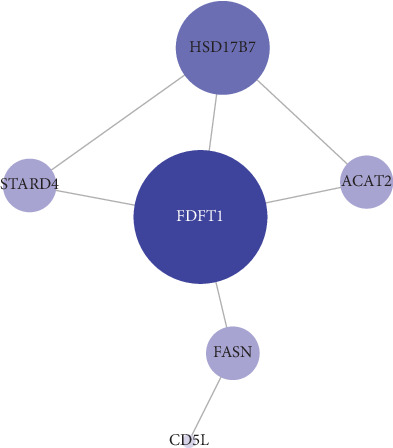
Protein-protein interaction analysis of age-related miRNA target genes in response to cholesterol and farnesyl diphosphate farnesyl transferase 1 (FDFT1) was identified as the key mRNA.

PPI analysis was performed on the 6 miRNA targets which included farnesyl diphosphate farnesyl transferase 1 (FDFT1), hydroxysteroid 17-beta dehydrogenase 7 (HSD17B7), steroidogenic acute regulatory protein-related lipid transfer domain-4 (STARD4), acetyl-CoA acetyltransferase 2 (ACAT2), fatty acid synthase (FASN), and CD5 antigen-like (CD5L). The interactions were visualized by the Cytoscape software, and the style of the figure was generated from statistics; to be specific, the size and color were influenced by the degree and the combined score dictated the edge size. It was designed so that low value led to small sizes and light colors. As is displayed in Figure 5, FDFT1 was identified as the key mRNA in the difference of response to cholesterol between aged and young macrophages.

## 4. Discussion

Impaired cholesterol metabolism has been discovered in senescent macrophages [4]. Although several studies have confirmed the relationship between altered cholesterol homeostasis in aged macrophages and AMD [4, 28], the miRNA-mRNA regulatory network is far from being fully understood. In this study, we sought to identify miRNA-mRNA interactions of macrophage aging and cholesterol-responsive difference between aged and young macrophages and then further analyzed the functional annotation and PPI of the miRNA targets. To the best of our knowledge, this is the first study to explore the miRNA-mRNA interactions aiming to get a better understanding of the pathological mechanism of AMD. Besides, our study is of significance for other lipid-related diseases of the elderly such as type 2 diabetes, cardiovascular disease.

Numerous mechanisms were found to be associated with macrophage aging through functional annotation of the differentially expressed miRNA targets. Among them, some have been reported to be related to AMD, including immunity [29, 30], inflammation [31, 32], chemotaxis [33, 34], angiogenesis [35, 36], oxidative stress [31, 37], and lipid metabolism [4, 28]. We further analyzed the impact of lipid dysregulation on aged macrophages by comparing aged and young macrophages which were treated with oxLDL or acLDL, because exogenous cholesterol plays a pathogenic role in promoting cholesterol dysregulation. In early AMD, lipid-rich drusen is a risk factor of disease progression; thus, our study on the influence of cholesterol on aged macrophages is significant to understand the lipid modulation role of macrophages in AMD progression.

We found that miR-714 was upregulated in aged murine peritoneal macrophages in response to cholesterol, and 6 miRNA-mRNA pairs were detected to play the role of skewing aged macrophages into a disease-promoting phenotype through abnormal lipid metabolism. MiR-714 has been reported to be upregulated in radiation-induced thymic lymphoma [38] and ischemia-reperfusion kidney injury [39] in mice. Besides, it has been reported that miR-714 is involved with vascular smooth muscle cell calcification by disrupting Ca2+ efflux proteins [40], suggesting that miR-714 may have a role in vascular homeostasis. According to miRTarBase [19], which is a database for experimentally validated microRNA-target interactions, it is currently known that miR-714 has less strong evidence pointing to Slc5a3, Wdr26, Ddr2, and Gprc5b through next-generation sequencing method. However, the role of miR-714 in macrophage aging or AMD pathogenesis has never been reported.

Among the 6 miRNA target genes, FDFT1, interacting with the other four genes, was the most significant one. FDFT1 encodes squalene synthase, which catalyzes the first committed step in cholesterol biosynthesis [41]. Biallelic pathogenic variants in FDFT1 will lead to squalene synthase deficiency, which is a rare inborn error of cholesterol biosynthesis with multisystem clinical manifestations including facial dysmorphism, nonspecific structural brain malformations, cortical visual impairment, and optic nerve hypoplasia [42]. FDFT1 has been reported to be related to sterol synthesis, which is expected to increase intracellular cholesterol and is associated with type 2 diabetes and coronary artery calcium [43]. FDFT1 has been found to be enriched in steroid biosynthesis pathway and upregulated in AMD by Zhao et al. [44]. They infer that FDFT1 may induce AMD by elevating the expression of cholesterol, which coincides with our results. Further studies should be conducted on miR-714-FDFT1, since modulation of cholesterol homeostasis may be a novel strategy for treating AMD.

## 5. Conclusion

Lipid metabolic process was found to play a significant role in both macrophage aging and response to cholesterol thus was regarded to be associated with the occurrence and progression of AMD. In addition, miR-714-FDFT1 may modulate cholesterol homeostasis in aged macrophages and have the potential to be a novel therapeutic target for AMD.

## Figures and Tables

**Table 1:** The top 9 Gene Ontology (GO) and the Kyoto Encyclopedia of Genes and Genomes (KEGG) Pathways of the differentially expressed miRNAs targets between aged and young macrophages.

Category	GO term	Description	Count	Genes	
BP	GO:0006955	Immune response	16	CCL24, CCL2, CXCR5, CXCL5, ENPP2, PRG4, CXCL13, H2-OB, MCPT4, CCL8, CMA1, TGTP2, CCL5, LTB, CCL7, BMPR1A	
	GO:0006954	Inflammatory response	10	CCL24, SELP, CCL2, CXCL5, CXCL13, EPHX2, CCL8, CD5L, CCL5, CCL7	
	GO:0006935	Chemotaxis	9	CCL24, CCL2, CXCR5, CXCL5, CXCL13, ENPP2, CCL8, CCL5, CCL7	
	GO:0045766	Positive regulation of angiogenesis	8	CCL24, PTGIS, CYP1B1, LRG1, SFRP2, HSPB1, CMA1, CCL5	
	GO:0055114	Oxidation-reduction process	8	CYP7B1, PTGIS, CYP1B1, SCD2, MAOA, CH25H, CP, DHCR24	
	GO:0070098	Chemokine-mediated signaling pathway	7	CCL24, CCL2, CXCL5, CXCL13, CCL8, CCL5, CCL7	
	GO:0071347	Cellular response to interleukin-1	7	LCN2, CCL24, CCL2, PTGIS, CCL8, CCL5, CCL7	
	GO:0006629	Lipid metabolic process^∗^	7	CYP7B1, PTGIS, SCD2, ENPP2, CH25H, EPHX2, DHCR24	
	GO:0008284	Positive regulation of cell proliferation	7	PRL2C3, CCND2, ENPP2, SFRP2, MZB1, PLAC8, TIMP1	

MF	GO:0005125	Cytokine activity	10	CCL24, CCL2, CXCR5, CXCL5, ENPP2, PRG4, CXCL13, H2-OB, MCPT4, CCL8, CMA1, TGTP2, CCL5, LTB, CCL7, BMPR1A	
	GO:0008009	Chemokine activity	7	CCL24, SELP, CCL2, CXCL5, CXCL13, EPHX2, CCL8, CD5L, CCL5, CCL7	
	GO:0005525	GTP binding	7	CCL24, CCL2, CXCR5, CXCL5, CXCL13, ENPP2, CCL8, CCL5, CCL7	
	GO:0016491	Oxidoreductase activity	7	CCL24, PTGIS, CYP1B1, LRG1, SFRP2, HSPB1, CMA1, CCL5	
	GO:0042803	Protein homodimerization activity	7	CYP7B1, PTGIS, CYP1B1, SCD2, MAOA, CH25H, CP, DHCR24	
	GO:0008201	Heparin binding	6	CCL24, CCL2, CXCL5, CXCL13, CCL8, CCL5, CCL7	
	GO:0005506	Iron ion binding	6	LCN2, CCL24, CCL2, PTGIS, CCL8, CCL5, CCL7	
	GO:0004497	Monooxygenase activity	4	CYP7B1, PTGIS, SCD2, ENPP2, CH25H, EPHX2, DHCR24	
	GO:0030414	Peptidase inhibitor activity	4	PRL2C3, CCND2, ENPP2, SFRP2, MZB1, PLAC8, TIMP1	

CC	GO:0005615	Extracellular space	29	GDF3, CCL2, CXCL5, ENPP2, LUM, IGFBP7, SERPINB1A, CCL8, CCL5, MMP3, CCL7, TIMP1, PRL2C3, CCL24, PTGIS, LRG1, MS4A1, CPA3, LTB, SELP, ACTA2, PRG4, SERPING1, LCN2, CXCL13, SFRP2, SERPINB2, HSPB1, CP	
	GO:0005576	Extracellular region	25	GDF3, CCL2, CXCL5, ENPP2, LUM, IGFBP7, CCL8, CCL5, MMP3, CCL7, TIMP1, CCL24, PRL2C3, PRG4, MZB1, SERPING1, CD5L, LCN2, BGN, PENK, CXCL13, SFRP2, SERPINB2, CMA1, CP	
	GO:0070062	Extracellular exosome	21	CPNE8, ACTA2, LUM, IGFBP7, EPHX2, SERPINB1A, SERPING1, CD5L, TIMP1, LCN2, CD38, CD55, ASPA, CD19, BGN, LRG1, MS4A1, HSPB1, CD79B, CP, VSIG4	
	GO:0009897	External side of plasma membrane	11	LY6A, FCER1A, LY6C1, SELP, CD55, CD19, CXCR5, MS4A1, CD79B, CD79A, BMPR1A	
	GO:0005789	Endoplasmic reticulum membrane	8	CYP7B1, PTGIS, CYP1B1, SCD2, CH25H, TGTP2, DHCR24, GIMAP1	
	GO:0031012	Extracellular matrix	7	BGN, LUM, IGFBP7, HSPB1, CMA1, MMP3, TIMP1	
	GO:0031225	Anchored component of membrane	4	LY6A, LY6C1, CD55, LY6D	
	GO:0031090	Organelle membrane	3	CYP7B1, CYP1B1, SCD2	
	GO:0019815	B cell receptor complex	2	CD79B, CD79A	

KEGG pathways	mmu04060	Cytokine-cytokine receptor interaction	10	CCL24, CCL2, CXCR5, CXCL5, CXCL13, CCL8, CCL5, LTB, CCL7, BMPR1A	
	mmu04062	Chemokine signaling pathway	8	CCL24, CCL2, CXCR5, CXCL5, CXCL13, CCL8, CCL5, CCL7	
	mmu05323	Rheumatoid arthritis	6	CCL2, CXCL5, H2-OB, CCL5, MMP3, LTB	
	mmu04640	Hematopoietic cell lineage	4	CD38, CD55, CD19, MS4A1	
	mmu00380	Tryptophan metabolism	3	KYNU, CYP1B1, MAOA	
	mmu04662	B cell receptor signaling pathway	3	CD19, CD79B, CD79A	
	mmu00120	Primary bile acid biosynthesis	2	CYP7B1, CH25H	

Abbreviations: GO: gene ontology; BP: biological process; MF: molecular functioning; CC: cellular component; KEGG pathways: Kyoto Encyclopedia of Genes and Genomes pathways; GTP: guanosine triphosphate.

**Table 2:** Gene Ontology (GO) and the Kyoto Encyclopedia of Genes and Genomes (KEGG) pathways of the differentially expressed miRNAs targets in response to cholesterol between aged and young macrophages.

Category	GO term	Description	Count	Genes
BP	GO:0006629	Lipid metabolic process	5	Stard4, Fdft1, Hsd17b7, Fasn, Acat2
	GO:0044255	Cellular lipid metabolic process	4	Stard4, Fdft1, Fasn, Acat2
	GO:0044281	Small molecule metabolic process	4	Fdft1, Hsd17b7, Fasn, Acat2
	GO:0008202	Steroid metabolic process	3	Stard4, Fdft1, Hsd17b7
	GO:0008610	Lipid biosynthetic process	3	Fdft1, Hsd17b7, Fasn
	GO:0044283	Small molecule biosynthetic process	3	Fdft1, Hsd17b7, Fasn
	GO:0055114	Oxidation-reduction process	3	Hsd17b7, Fasn, Acat2
	GO:0097384	Cellular lipid biosynthetic process	2	Fdft1, Fasn
	GO:0006695	Cholesterol biosynthetic process	2	Stard4, Fdft1
	GO:0008203	Cholesterol metabolic process	2	Fdft1, Hsd17b7
	GO:0030258	Lipid modification	2	Stard4, Acat2
	GO:0006631	Fatty acid metabolic process	2	Fasn, Acat2

MF	GO:0016407	Acetyltransferase activity	2	Fasn, Acat2
	GO:0016616	Oxidoreductase activity, acting on the CH-OH group of donors, NAD or NADP as acceptor	2	Hsd17b7, Fasn

KEGG pathways	mmu01100	Metabolic pathways	4	Fdft1, Hsd17b7, Fasn, Acat2
	mmu01212	Fatty acid metabolism	2	Fasn, Acat2
	mmu00100	Steroid biosynthesis	2	Fdft1, Hsd17b7

Abbreviations: GO: gene ontology; BP: biological process; MF: molecular functioning; KEGG pathways: Kyoto Encyclopedia of Genes and Genomes pathways; NAD: nicotinamide adenine dinucleotide; NADP: nicotinamide adenine dinucleotide phosphate.

## Data Availability

All raw data in this article can be obtained by emailing the corresponding author.
